# Correction: Magnetic sodium alginate grafted with waste carbonaceous material for diclofenac sodium removal: optimization of operational parameters and process mechanism

**DOI:** 10.1039/d4ra90014f

**Published:** 2024-02-21

**Authors:** Salhah D. Al-Qahtani, Saham Ibarhiam, Sahar Sallam, Awatif R. Z. Almotairy, Ameena M. Al-bonayan, Alaa M. Munshi, Nashwa M. El-Metwaly

**Affiliations:** a Department of Chemistry, College of Science, Princess Nourah Bint Abdulrahman University P. O. Box 84428 Riyadh 11671 Saudi Arabia nmmohamed@uqu.edu.sa n_elmetwaly00@yahoo.com; b Department of Chemistry, College of Science, University of Tabuk 71474 Tabuk Saudi Arabia; c Department of Chemistry, Faculty of Science, Jazan University P. O. 45142 Jazan Saudi Arabia; d Department of Chemistry, Faculty of Science, Taibah University Yanbu 30799 Saudi Arabia; e Department of Chemistry, Faculty of Applied Science, Umm Al Qura University Makkah 24230 Saudi Arabia; f Department of Chemistry, Faculty of Science, Mansoura University El-Gomhoria Street 35516 Egypt

## Abstract

Correction for ‘Magnetic sodium alginate grafted with waste carbonaceous material for diclofenac sodium removal: optimization of operational parameters and process mechanism’ by Salhah D. Al-Qahtani *et al.*, *RSC Adv.*, 2023, **13**, 6466–6480, https://doi.org/10.1039/D3RA00495C.

The authors would like to clarify the affiliation list of the published article to correctly show where the work was conducted. In the original article, the affiliation at the time of publication of two authors (Ameena M. Al-bonayan and Alaa M. Munshi) was listed as Department of Chemistry, Faculty of Science, Mansoura University, El-Gomhoria Street, Egypt. However, the work was conducted at the Department of Chemistry, Faculty of Applied Science, Umm Al Qura University, Makkah 24230, Saudi Arabia. The corrected list of affiliations at the time of publication of the original article is presented here.

The Royal Society of Chemistry apologises for these errors and any consequent inconvenience to authors and readers.

## Supplementary Material

